# Clinical utility of intraoperative direct wave monitoring in patients with pre-operative motor deficits: Case series

**DOI:** 10.1016/j.ijscr.2023.109164

**Published:** 2023-12-15

**Authors:** Shyam Duvuru, Vivek Sanker, Maneeth Mylavarapu, Sejal Lund, Rahul Jena, Tirth Dave

**Affiliations:** a Department of Neurosurgery, Apollo Specialty Hospitals, Madurai, Tamil Nadu, India; b Department of Neurosurgery, Trivandrum Medical College Hospital, Trivandrum, India; c Department of Public Health, Adelphi University, New York, USA; d Department of Internal Medicine, Shaheed Mohtarma Benazir Bhutto Medical College, Karachi, Pakistan; e Department of Internal Medicine, Bharati Vidyapeeth Medical College, Pune, India; f Bukovinian State Medical University, Chernivtsi, Ukraine

**Keywords:** Intraoperative neuromonitoring, IONM, MEP, D wave, SSEP, EMG, Spine surgery

## Abstract

**Introduction and importance:**

D-wave (Direct waves) are Motor Evoked Potentials (MEPs) generated by a single transcranial stimulation and captured by attaching an epidural recording electrode caudal to the vulnerable area. Intraoperative neurophysiologic monitoring (IONM) is widely used in neurosurgery to recognize important neurological structures but can be challenging in the pediatric population due to incomplete neural development.

**Case presentation:**

Case 1: A 48-year-old female presented to the outpatient department with complaints of difficulty walking for the past six months, numbness and weakness in bilateral lower limbs with recurrent falls for the past 1 month. Case 2: A 12-year-old boy presented to the emergency room with a history of inability to use both upper and lower limbs on the right side with tremulousness.

**Clinical discussion:**

Magnetic resonance imaging (MRI) Spine in the first case revealed a D9-D10 calcified meningioma with significant spinal cord compression. In the second case, MRI Spine showed C1-C2 Intramedullary Space Occupying Lesion (SOL) and was planned for C1-C2 laminectomy with midline myelotomy. The first case was planned for microsurgical excision of the lesion under IONM guidance. The procedure went smoothly. Microsurgical gross total resection (GTR) of the intramedullary SOL under IONM Guidance was done for the second case. Postoperatively, the first patient showed no neurological compromise or complications. In the second case, following surgery, the child recovered gradually from surgery.

**Conclusion:**

This case series demonstrates the successful surgical management of two cases of spinal cord tumors through an IONM-guided surgery and the effective use of D waves in such challenging cases.

## Highlights

• Intraoperative D-wave monitoring offers a valuable tool for spinal cord tumor resection, in cases with pre-operative motor deficits and the need for long-term motor function prediction.
• The study demonstrates successful application of D-wave monitoring in two cases of spinal cord tumor resection, with favorable post-operative outcomes.
• D-wave recordings, in conjunction with other modalities such as MEPs and SSEPs, provide a comprehensive assessment of spinal pathway functioning during surgery.
• D-wave monitoring proves its capability in predicting long-term motor performance, aiding in patient recovery and rehabilitation planning.
• While D-wave monitoring offers significant advantages, challenges such as reduced signals and required expertise in interpreting data should be considered by surgeons.

## 1 Introduction

Due to the close proximity and the abundance of functionally diverse white matter tracts, spinal cord tumors present various surgical challenges than their supratentorial counterparts. Primary intramedullary spinal cord tumors (IMSCTs) originating in the spinal cord parenchyma are perhaps the most difficult to treat since their removal might cause post-operative functional deficits from fiber manipulation and pose a danger to patient quality of life [1]. Intraoperative neurophysiological monitoring (IONM) techniques have been developed and widely used in recent years to reduce the risk of iatrogenic harm by real-time evaluation of neural circuit integrity [2]. Numerous IONM modalities have been created to simultaneously monitor at-risk pathways and are frequently used in conjunction with one another. One of the spinal channels implicated in musculoskeletal regulation, the lateral corticospinal tract (CST), can be continuously monitored using direct waves (D-waves) [3,4]. It is a common practice to monitor somatosensory-evoked potentials (SSEP), which indicate ascending cuneatus and gracilis pathways, and motor-evoked potentials (MEP), which correspond to descending motor neurotransmission [5].

Due to the difficulties in predicting neurological deterioration in the immediate post-operative period, MEPs have historically been preferred over D-waves. Recent long-term investigations, however, have demonstrated the capability of D-waves to predict motor performance over the long term [6]. In combination with SSEP and MEPS, the gold standard for CST monitoring, D-waves are increasingly used during tumor removal to produce a comprehensive picture of distinct yet connected spinal pathway functioning [7,8]. Here, we report two cases of spinal cord tumors, which were successfully resected with favorable post-operative outcomes with the application of D waves.

## 2 Case report

### 2.1 Case - 1

**Fig. 1:**
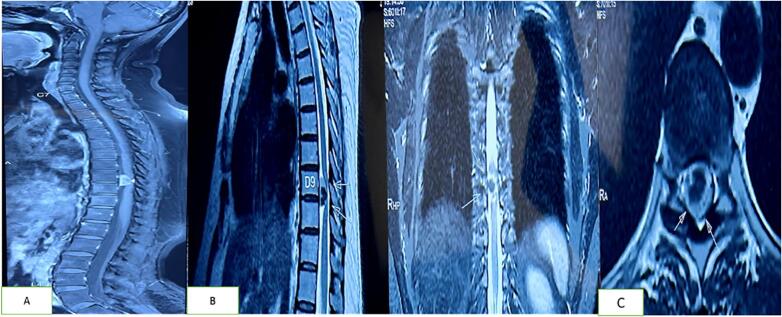
Magnetic Resonance Imaging (MRI) of the spine showing a well-defined intradural extramedullary lesion arising at D9 level with significant cord compression and myelomalacia. The lesion appears to be variable in consistency with outer calcification.

A 48-year-old female presented to the outpatient department with complaints of difficulty in walking for the past six months, numbness and weakness in bilateral lower limbs (right more than left) with recurrent falls for the past 1 month. The patient was a known diabetic and hypertensive, was on regular medication, and had no documented drug allergies. Her general examination was unremarkable except for lower limb weakness, right side 3/5 and left side 4/5, and numbness of the lower limbs (right > left), with no bowel or bladder disturbances. All the baseline pre-operative investigations were normal. Magnetic resonance imaging (MRI) of the spine revealed a D9-D10 calcified meningioma with significant spinal cord compression (Fig. 1). She was planned for microsurgical excision of the lesion.

After positioning the patient in prone, a midline vertical skin incision was made, it was deepened, and sub-periosteal muscle dissection was done. The laminae were excised, and then dura was exposed. Under the microscope, durotomy was done, and D wave recordings were noted and obtained. D9 nerve root was transected, and then the arachnoid plane was developed. The tumor was greyish-white, firm to hard in consistency, and well-defined. It was originating from the ventral dura. The plane between the tumor and the spinal cord was present. The tumor was debulked after disconnecting from the ventral dura. The entire tumor was resected using a cavitron ultrasonic surgical aspirator (CUSA), and then hemostasis was achieved using Surgicel. Dura was closed using a 4-0 prolene. Hemostasis was achieved, and a drain was placed. Wound closure was done in layers.

**Fig. 2:**
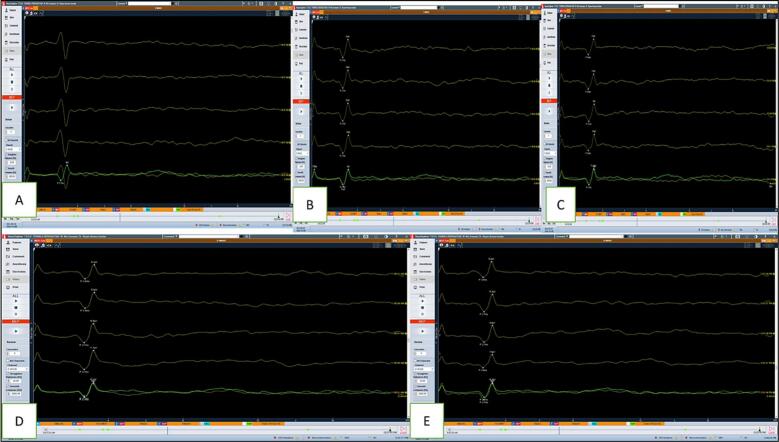
D-wave monitoring in patient 1: A: D-wave recorded from proximal to the tumor (i.e., towards the cranial side); B: D-wave recorded distal to the tumor (i.e., towards the caudal side); C, D, E: D-wave recordings during the surgery.

**Fig. 3:**
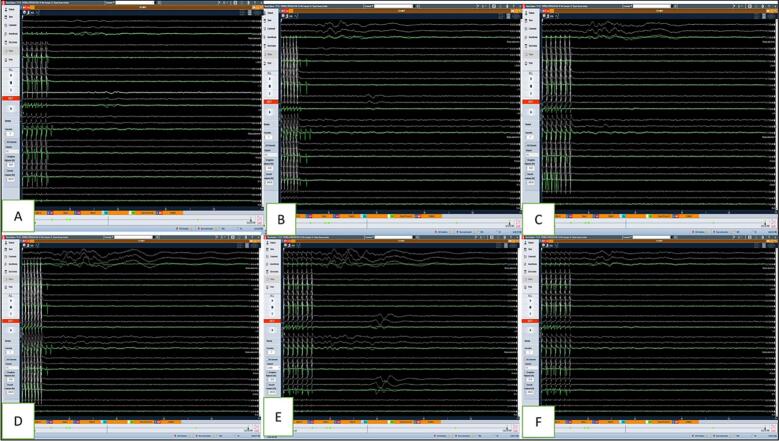
MEP monitoring in patient 1: A: baseline MEP; B: MEP during the surgery; C, D: increase in the baseline MEP current by 10 %, yet left abductor hallucis wave - absent; E: increase in the baseline MEP current by 10 %, with left abductor hallucis (AH) and right tibialis anterior (TA) muscles waves (circled); F: post-operative return to baseline.

The IONM during the surgery was almost stable, with a 10 % baseline increase in MEP during the surgery, and the wave potentials returned back to pre-operative baseline readings. Detailed D-wave and MEP monitoring findings are reported in Fig. 2, Fig. 3.

The patient was extubated on the table. Immediately after surgery, her power on the right side was 0/5, and on the left side was 3/5. On the third post-operative day, she was mobilized to wheelchair. Physiotherapy and rehabilitation were initiated. On histopathological examination, the excised tumor was found to be grade I Meningioma. At the time of discharge, the patient was afebrile, power in the right lower limb was 1/5, left lower limb was 4/5, bladder drained via Foley catheter, and the operated site was healthy. At one month follow-up, her power in the right lower limb was 4/5, and her left lower limb was 5/5. She was able to walk independently and regained her bladder and bowel sensations. At 6th month follow up she had regained her power completely and it was 5/5 in both the lower limbs.

### 2.2 Case - 2

A 12-year-old boy presented to the emergency room with a history of inability to use both upper and lower limbs on the right side with tremulousness for the past 3 weeks which has worsened since 2 days. Initially he had difficulty in using his right upper limb in the form of inability to write using his pen and this progressed to the left upper limb also. Over the last two days, he has difficulty in walking to the classroom and also has tremulousness in his lower limbs. The patient had no history of fever, myalgia, headache, loss of consciousness, and visual disturbances. On examination, right upper limb power was 2/5, and lower limb power was 3/5, with no deficits on the left side. There was a wasting of the small muscles of the hand on the right side. There were no bowel or bladder disturbances. He underwent an MRI of the Spine and was diagnosed with C1-C2 Intramedullary Space Occupying Lesion (SOL), which was planned for microsurgical gross total resection (GTR) of the intramedullary SOL under IONM Guidance.

**Fig. 4:**
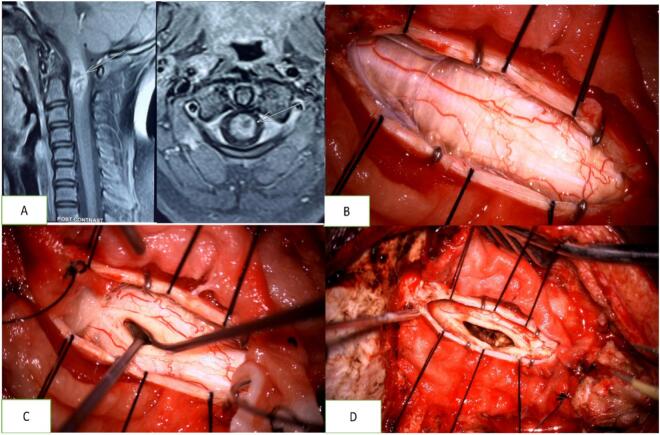
A: MRI of the cervical spine and cranio-vertebral junction showing an ill-defined intramedullary lesion with surrounding edema and irregular contrast uptake. B, C, D: Intraoperative images showing the dural opening, myelotomy, tumor, and tumor bed.

With the patient in prone position and head held in a Mayfield three-pin head holder, a midline vertical skin incision was made from just below the external occipital protuberance to the C4 level, and sub-periosteal muscle dissection was done. Laminae of C1 and C2 were removed to expose the dura. Under microscope, durotomy was performed, arachnoid was cut, and CSF was let out (Fig. 4).

**Fig. 5:**
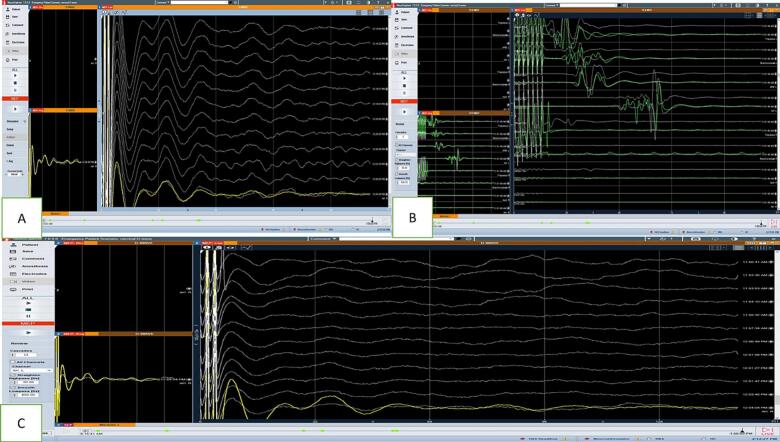
D-wave monitoring in patient 2: A: baseline D-wave; B: significant drop in D-wave amplitude in left trapezius (almost 40 %) during dissection of the tumor; C: D-wave recordings during the surgery.

**Fig. 6:**
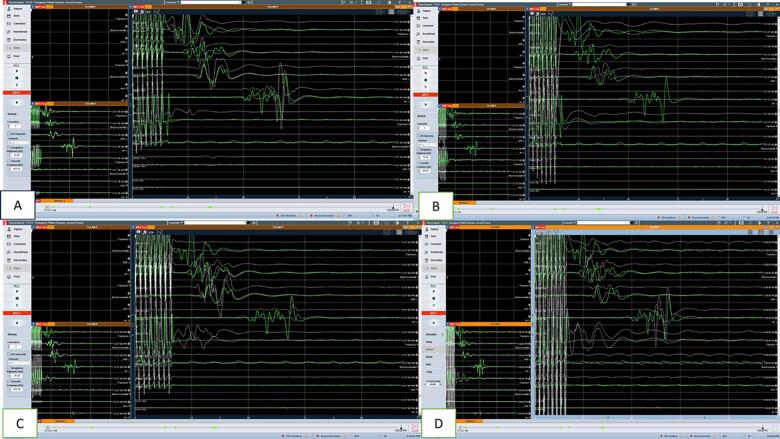
MEP monitoring in patient 2: A: baseline MEP; B, C: significant drop (40–50 %) in the MEP amplitude in left lower limb (circled); D: post-operative MEP (almost recovered).

The multi-modality monitoring was employed on the patient, including TcMEP and SSEP of bilateral upper and lower limbs. The stimulated EMG was recorded in bilateral upper limbs. The D wave recording was also done, and baseline recordings showed no right-side signals. During laminectomy and tumor excision, the MEPs were constantly monitored. Myelotomy was performed, and the tumor-cord interface was dissected. The tumor was debulked using CUSA and then gradually dissected off the cord surface. There was a significant drop in MEP and D-wave amplitude during surgery on the left side. After 30 min, the MEPs and D-waves started showing signs of recovery. There was a drop in left trapezius potentials by more than 40 % during dissection. During the final dissection, there was a bleed from the ventral surface and a simultaneous drop in D wave and MEP. Bleeding was arrested using bipolar cautery. After 20 min and a warm saline wash, the D wave recovered to baseline. Left upper limb MEPs improved and left lower limb MEPs showed improvement by 30 % from the drop. At the end of the surgery, the MEPs and D-wave amplitude recovered almost equal to the pre-op baseline reading. The dura was closed, and hemostasis was achieved with fibrin sealant. A drain was placed, and the wound was closed in layers. A comprehensive description of the D-wave and MEP monitoring findings is outlined in Fig. 5, Fig. 6**.**

Postoperatively, the child was not able to be weaned from the ventilator due to weakness of the respiratory muscles. His power on the right side was 0/5, and on the left side was 3/5. He underwent percutaneous tracheostomy on the 3rd post operative day and was slowly weaned off to BIPAP support on the 16th post operative day. Finally, at discharge, he was conscious and oriented, and power in both his right upper and lower limbs was found to be 4/5, and his left side was 5/5. The histopathology was Grade II Astrocytoma. He was weaned off tracheostomy on the 35th post-operative day. After rehabilitation, he was able to walk without support and was performing his activities of daily living for 2 months. At 6 months follow up he was able to write normally, comb his hair and button his shirts without any help. He is able to walk without support and his bowel bladder was continent.

## 3 Discussion

The advent of invasive spinal surgery came with its own unique challenges. Assessing postoperative and long-term neurological sequelae has become imperative and has a direct influence on patient safety and outcomes. The term “intraoperative neurophysiological monitoring” (IONM) describes the use of electrophysiological techniques to check on the structural and functional integrity of neural structures during surgery. A wide range of consequences of post-operative neurological injuries can be reduced by intraoperative neuromonitoring (IONM). The goal is to detect potential post-operative neurological deficits by continuously or intermittently monitoring and assessing neural structures, preventing damage, and preserving the functionality of the nervous, and this can be achieved by selecting appropriate monitoring techniques based on basic anatomical and physiological principles while taking surgical and anesthetic considerations in order to improve IONM diagnostic performance [7,[9], [10], [11]].

Routine IONMs used in spinal surgeries include somatosensory evoked potentials (SSEPs), motor evoked potentials (MEPs), D-waves, and electromyography as neuromonitoring modalities [12]. D waves result from direct single stimulation of the motor cortex and remain the single most effective measure of long-term motor function when compared to its counterparts such as EMG, MEPs, and SSEPs [9]. D waves and epidural MEPs play an important role in evaluating long-term motor function and patient outcomes, as seen in our cases. Existing literature agrees with their efficacy with respect to evaluating long-term motor function and patient outcomes, especially when they are used synergistically in both pediatric and adult populations [[9], [10], [11]]. Advantages and disadvantages of various IONMs are discussed in Table 1 [12].

**Table 1:** Summary of advantages and disadvantages of IONM modalities.

Types of IONM	Advantages	Disadvantages
SSEPs	▪ Noninvasive ▪ High sensitivity & specificity ▪ Continuous monitoring throughout the surgery ▪ It can be used in conjunction with neuromuscular blockade ▪ Good for detecting spinal cord ischemia compression and traction of the spinal cord	▪ Inability to monitor individual nerve root function effectively. ▪ Potential temporal summation delay of up to 16 min in the detection of a signal change ▪ Not a direct corticospinal tract monitor. ▪ Cannot detect motor injury or anterior spinal artery syndrome.

MEPs		
Muscle MEPs	▪ Extremely capable of identifying post-operative motor impairments. ▪ More effective for monitoring because they don't need to be averaged and can be repeated at a rate of 0.52 Hz. ▪ Has a greater MEP generation rate and doesn't need an epidural electrode. ▪ Compared to SSEPs and D Waves, it helps in earlier diagnosis of spinal cord ischemia	▪ Primarily monitor for corticospinal tract integrity hence other spinal cord components could be compromised without recognition. ▪ Neuromuscular blockade prohibited ▪ Quality and interpretation of mMEP recordings can be affected by anesthesia ▪ In rare cases provoke a seizure, especially in patients with a pre-existing seizure disorder. ▪ Cannot be continuously monitored ▪ Some patients with deep brain stimulators or cochlear implants should not receive it. ▪ Rarely, muscle MEPs have been linked to a variety of negative outcomes, including intraoperative consciousness, tongue or lip laceration, mandibular fracture, cardiac arrhythmias, epileptic seizures, and scalp burns. ▪ When pre-operative motor impairments are present at the time of surgery, the likelihood of obtaining MEPs is greatly reduced. ▪ Due to incomplete motor pathway maturation, this procedure is often difficult in patients under 6 years old.
D waves	▪ Monitored continuously during the surgery, providing real-time information about the functional integrity of the motor pathways ▪ Resistant to volatile anesthetics, providing stable signals in surgeries that require heavy sedation ▪ Allows to Proceed even with MEP/SSEP Loss ▪ Greater predictive value for post-operative deficits ▪ Provide relevant data regarding long-term motor outcomes, even in compromised patients. ▪ Remain stable unless there is a severe, near-total injury to the corticospinal tract. This characteristic makes them less likely to produce false positives due to minor or moderate injuries. ▪ Receptive to Single Pulse Stimulation and rapid acquisition ▪ The spinal electrode can be moved throughout the intervertebral spaces to determine the lesion level. ▪ Due to their accuracy in predicting long-term motor status, they are regarded as the gold standard for motor pathway monitoring during intramedullary spinal cord tumor surgery.	▪ Don't provide information on sensory conduction ▪ May increase the cost and duration of the surgical procedure ▪ Limitations in pediatric patients, generally under the age of 4, due to the incomplete maturation of motor pathways ▪ D waves can only be used in regions of the spine above T10–T11 with an epidural recording electrode directly over the spinal cord. ▪ The rotation of the corticospinal tract in relation to the recording electrode during spinal curvature correction causes inaccuracy in some surgeries, such as scoliosis surgery. ▪ Lack of Laterality Distinction ▪ The placement of electrodes may be compromised by previous scarring

EMGs		
t-EMG	▪ High sensitivity for detecting breaches in the medial pedicle wall during surgery ▪ Useful in minimally invasive surgery when it may be difficult to see anatomical landmarks ▪ Simple to use and understand ▪ Specifically used in spine surgery to confirm the accurate placement of pedicle screws ▪ During tethered cord surgeries, stimulating various structures along with t-EMG monitoring is valuable in determining if functional neural elements are present	• Nerve conduction may be hampered by a pre-operative nerve-root deficiency, requiring greater stimulation thresholds. • Sensitive to a large number of anesthetics • t-EMG primarily monitors motor nerves and thus may not provide comprehensive monitoring of all neural structures at risk during surgery
s-EMG	▪ Continuous monitoring throughout the surgery ▪ It allows for early detection of nerve irritation or injury during surgical procedures, potentially reducing post-operative motor deficits.	▪ The use of neuromuscular blockade is forbidden ▪ Sensitive to variations in temperature ▪ Alerts that are frequently false positives

IONM, intraoperative neurophysiological monitoring; SSEP, somatosensory sensory evoked potential; MEPs, motor-evoked potentials; D wave, direct wave; EMG, electromyography; sEMG, spontaneous EMG; tEMG, triggered EMG.

Shigematsu et al. conducted a propensity-matched analysis and reported that although the rescue rate (an indicator of detecting and intervening in reversible spinal cord injury) did not significantly differ between TES-MEPs with D-wave and TES-MEPs without D-wave, there was a significant reduction in false-positivity rate within the TES-MEPs-with-D-wave group [13]. D-wave recording has been shown to remain stable even in cases of MEP/SSEP loss. D wave monitoring has a high specificity and consistency over time compared to MEPs, with the specificity being more than twice as high [6]. Additionally, in patients with pre-existing motor deficits, MEPs show poor recordability, while D wave monitoring remained a good modality in such a scenario [10,14], similar to our cases. A study by Bir et al. corroborated this using D waves and MEP intraoperative monitoring in 31 patients with pre-existing motor deficits and found that they could be recorded in 100 % and 79.4 % of patients [15].

Although multimodal IONM (SSEP, MEP, and D-wave monitoring) significantly predicted postoperative deficits, D-wave recordings have been found to be more predictive of long-term outcomes rather than short-term outcomes, emphasizing the importance of correlating the time point of measurement with its predictive value [6,7].

In the study conducted by Bir et al., immediately after surgery, m-MEP demonstrated perfect sensitivity (100 %), while D-wave exhibited high specificity (100 %) and positive predictive value but lower sensitivity (14.2 %). These findings indicate that m-MEP alarms during surgery closely aligned with immediate postoperative outcomes in terms of sensitivity, while D-wave had superior specificity [15]. An intact D-wave signal amid decreased m-MEP amplitudes effectively anticipates the recovery of transient deficits, serving as a reliable predictor for long-term neurological status [10,14]. Similar findings were seen in Case 1, where the patient made a full recovery one-month post-surgery. Studies by Ghadirpour et al. and Costa et al. found better relative recordability for D waves (94.1 % and 92.2 %, respectively) [7,16]. Of note, in spite of unavailable histopathological evidence and previous motor dysfunction in our second case, we were still able to use D waves to successfully monitor the patients intraoperatively.

D wave and MEP monitoring, despite all these advantages, are still far from being considered the perfect IONMs. In patients with pre-existing motor deficits, prior surgery or radiotherapy, or an intramedullary mass, in which CST conduction velocity may be directly affected, inconsistent or reduced signals can occur, thereby altering D-wave patterns [15]. Additionally, D-wave neuromonitoring is not applicable when the spinal tumor is situated below the T10-T11 level of the spinal cord since there is no corticospinal tract (CST) input below this level, resulting in the absence of detectable signals [4]. With regards to MEPs, due to their low specificity, they can't be effective in predicting long-term outcomes. Furthermore, MEPs are more subjected to signal attenuation by anesthesia when compared to D waves, owing to their polysynaptic origin [17,18]. Hence, on the surgeon's part, there is a need for expertise in interpreting signal/noise ratios and troubleshooting noise artifacts due to small amplitudes of D waves when compared to limb MEPs.

This case series was reported in line with the PROCESS guidelines [19].

## 4 Conclusions

In both patients, the intraoperative data pointed to a post-operative improvement in motor condition. D-wave monitoring is a crucial tool in the broader perspective of neurosurgery and neurophysiological monitoring owing to its ability to function in patients with pre-operative motor defects, resistance to general anesthesia, and muscle relaxants. Although the major goal of IONM is to prevent any iatrogenic harm, an intraoperative improvement is of the utmost importance for accurate planning of a rehabilitation strategy as well as from a prognosis standpoint.

## Abbreviations

IMSCT: Intramedullary spinal cord tumors
IONM: Intraoperative neurophysiological monitoring
CST: Corticospinal tract
D wave: Direct wave
SSEP: Somatosensory-evoked potentials
MEP: Motor-evoked potentials
MRI: Magnetic resonance imaging
CUSA: Cavitron ultrasonic surgical aspirator
SOL: Space Occupying Lesion (SOL)
GTR: Gross total resection

## Consent for adult patient

Written informed consent was obtained from the patient for publication and any accompanying images. A copy of the written consent is available for review by the Editor-in-Chief of this journal on request.

## Consent for pediatric patient

Written informed consent was obtained from the patient's parents/legal guardian for publication and any accompanying images. A copy of the written consent is available for review by the Editor-in-Chief of this journal on request.

## Ethical approval

Ethical approval was not required for the case report as per the country's guidelines.

## Funding

No funding was acquired for this study.

## CRediT authorship contribution statement

All the authors contributed equally in drafting, editing, revising, and finalizing the case report.

## Guarantor

Shyam Duvuru.

## Declaration of competing interest

None declared.

## Data Availability

The data supporting this article's findings are available from the corresponding author upon reasonable request.
